# Application of functional magnetic resonance imaging in identifying responsible brain regions associated with spinal diseases related pain

**DOI:** 10.3389/fmed.2025.1585799

**Published:** 2025-11-20

**Authors:** Jing Zhang, Nannan Wang, Le-Meng Ren, Xiaopei Sun, Jun-Peng Zhang, Yuehuan Zheng

**Affiliations:** 1 Department of Radiology, Ruijin Hospital, Shanghai Jiao Tong University School of Medicine, Shanghai, China; 2 Department of Nursing, Ruijin Hospital, Shanghai Jiao Tong University School of Medicine, Shanghai, China; 3 Department of Orthopaedics, Ruijin Hospital, Shanghai Jiao Tong University School of Medicine, Shanghai, China; 4 Department of Rehabilitation, Shanghai Ruijin Rehabilitation Hospital, Shanghai, China; 5 School of Rehabilitation Science, Shanghai University of Traditional Chinese Medicine, Shanghai, China

**Keywords:** brain map, spinal diseases related pain, responsible brain region, functional magnetic resonance imaging, brain remodeling

## Abstract

**Background:**

Spinal diseases related pain represents a critical clinical issue that demands urgent resolution. Current treatment and assessment strategies predominantly focus on peripheral mechanisms. The application of functional magnetic resonance imaging (fMRI) offers a promising approach to identifying potential central targets for intervention.

**Methods:**

We retrospectively included 31 patients with spinal diseases related pain and 32 controls with non-spinal, orthopedic complaints (no chronic neurological or psychiatric disorders). All participants underwent resting-state brain fMRI (eyes closed, awake). We quantified amplitude of low-frequency fluctuations (ALFF) with mean normalization (mALFF) and z-transformation (zALFF), regional homogeneity (ReHo; 27-voxel neighborhood), seed-based functional connectivity (FC; pre/postcentral seeds), and degree centrality (DC; binary and weighted). Between group tests used voxel-wise two-sample t_tests with Gaussian random field (GRF) correction.

**Results:**

Patient group was associated with increased m/zALFF in right cerebellar lobule IX and right Superior Frontal Gyrus, medial part, and lower activity in bilateral postcentral gyri and the cuneus, decreased m/zALFF in bilateral postcentral gyri. ReHo analysis confirmed reduced local synchrony in postcentral regions, spatially overlapping with ALFF findings. FC analyses revealed enhanced cerebellar-thalamic connectivity (Crus1/2, thalamus) but reduced connectivity in sensorimotor and higher-order cortical networks. DC showed hyperconnectivity in left cerebellar Crus I with reduced Superior Frontal Orbital (Frontal_Sup_Orb). All findings survived GRF correction at the pre_specified thresholds.

**Conclusion:**

Resting-state brain fMRI indicates a cerebello-thalamo-cortical alteration pattern in spinal diseases related pain featuring cerebellar involvement, prefrontal subspecialization, and multilevel sensorimotor disruption. These cross-sectional associations may inform hypothesis-generation for future neuromodulation studies and provide candidate biomarkers for monitoring, pending prospective validation.

## 1 Introduction

Spinal diseases related pain is a leading cause of disability and health-care expenditure worldwide (1, 2). Importantly, the term “spinal diseases” refers to clinically defined conditions (e.g., disc degeneration, spinal stenosis, spondylosis), whereas commonly cited lifetime prevalence figures (e.g., for non-specific low back pain) describe a symptom rather than a diagnosis (3, 4). To avoid conflation, here we focus on patients with symtoms of spinal diseases related pain.

Spinal diseases related pain is traditionally managed through peripheral interventions such as pharmacotherapy (5, 6), physical therapy (7), or surgery (8, 9), etc. However, these approaches often fail to address central nervous system alterations increasingly recognized in chronic pain states (10–12). Resting-state fMRI has emerged as a valuable tool to identify central biomarkers, including ALFF (13–17), ReHo (15, 17, 18), and functional connectivity (10, 11, 19, 20). Recent studies have reported brain network changes (21–26), particularly in sensorimotor, limbic, and thalamo-cortical circuits, highlighting the need for a shift toward centrally focused models of spinal pain pathophysiology.

This study used fMRI to investigate the brain remodeling mechanisms of spinal diseases related pain and to identify the specific brain mapping patterns involved in pain processing pathways. The primary objective was to establish a comprehensive evaluation framework for assessing responsible brain region and connection alterations in patients with spinal diseases related pain. By implementing a central-peripheral integrated assessment system, this research aims to provide a robust scientific foundation for enhancing diagnostic accuracy, optimizing therapeutic interventions, and improving prognostic evaluation in the management of spinal diseases related pain (5–9, 27, 28).

## 2 Methods

### 2.1 Study design and participants

This retrospective study enrolled participants who underwent functional magnetic resonance imaging (fMRI) examinations at the Department of Orthopedics, Ruijin Hospital, Shanghai Jiao Tong University School of Medicine, between October 2023 and October 2024. The study protocol was approved by the Institutional Review Board of Ruijin Hospital (Ethical Approval Number: 20240902113233506). This retrospective study included two groups: (i) patients with symptoms of spinal diseases related pain due to clinically diagnosed spinal pathology (e.g., disc degeneration, osteoporotic fracture, stenosis), and (ii) controls who presented with non-spinal orthopedic complaints and no history of chronic neurological or psychiatric disorders. All participants were right-handed and underwent brain fMRI on the same 3.0-T scanner within the same institutional protocol. Major exclusion criteria for both groups were prior spinal surgery, major neurological disease (e.g., stroke, traumatic brain injury, neurodegeneration), major psychiatric illness, claustrophobia, unstable systemic disease, or incomplete records. Pain intensity (VAS) was extracted from clinical records closest to the scan date.

### 2.2 fMRI acquisition

All data were acquired on a GE 3.0-T system. Participants lay supine, eyes closed, relaxed but awake. Resting-state functional images used gradient-echo EPI with the following parameters: TR/TE = 2000/30 ms, flip angle = 90°, 43 axial slices, interleaved order, slice thickness = 3.2 mm (voxel 3.4 × 3.4 × 3.2 mm^3^), matrix 64 × 64, FOV 220 × 220 mm^2^, 240 volumes, parallel acceleration = 2. Anatomical T1-weighted images used a 3D SPGR sequence: TR/TE = 8100/3.1 ms, flip angle = 8°, 176 sagittal slices, isotropic 1 mm^3^ voxels, FOV 256 × 256 mm^2^. The first 10 rs-fMRI volumes were discarded to allow signal stabilization.

### 2.3 Preprocessing and first-level metrics

Preprocessing was performed in RESTplus (29)(SPM12-based) on MATLAB R2013b. Steps included slice-timing correction; rigid-body realignment (subjects with >3 mm translation or >3° rotation were excluded); normalization to MNI152 template; and nuisance regression (24-parameter motion, white matter, CSF). Data were band-pass filtered at 0.01–0.08 Hz. ALFF was computed and expressed as mean-normalized (mALFF) and z-standardized (zALFF) maps within a gray matter mask (30). ReHo (31) was computed using Kendall’s coefficient over a 27-voxel (3 × 3 × 3) neighborhood and then spatially smoothed with a 6 mm FWHM Gaussian kernel.

T1-weighted structural MRI provided the anatomical reference for EPI-anatomical registration, MNI normalization, tissue segmentation (GM/WM/CSF) for nuisance modeling, and ROI/surface definitions.

### 2.4 Functional connectivity and degree centrality

Seed-based functional connectivity (FC) analyses used bilateral precentral and postcentral gyri as *a priori* regions of interest due to their established roles in pain-related sensorimotor processing and representations of nociceptive input. Seed time series were correlated with whole-brain voxels and Fisher-z transformed. Degree centrality (DC) was computed in both binary and weighted forms using RESTplus defaults (voxelwise correlation matrix thresholding), providing complementary indices of network hubness. Exact parameter settings are reported to facilitate replication.

### 2.5 Group-level statistics

Between group comparisons employed voxel-wise two-sample *t*_tests in SPM 12(32, 33)with age and sex as covariates. Multiple comparisons were controlled with Gaussian Random Field (GRF) correction at the pre-specified thresholds (ALFF/ReHo/FC: voxel *p* < 0.01, cluster *p* < 0.01; DC: voxel *p* < 0.05, cluster *p* < 0.05, matching the original analysis). Clinical variables were summarized as mean ± SD and compared with *t*-tests or *χ*^2^ tests as appropriate (two-tailed *p* < 0.05) by SPSS (version 26.0; IBM, Armonk, NY, United States) statistical software.

## 3 Results

### 3.1 Participant characteristics

Thirty-one spinal diseases related pain patients (15 males/16 females; 64.52 ± 16.40 years) and 32 controls (9 males/23 females; 47.69 ± 13.45 years) were included after quality control. Groups did not differ in sex distribution (*p* > 0.05); VAS pain scores were higher in patient group (*p* < 0.001). Age was included as a covariate in imaging analyses. The demographic and clinical characteristics of patients are shown in Table 1.

**Table 1:** Participant characteristics.

Variables	Patients (*n* = 31)	Controls (*n* = 33)	*p* value
Age(y)	64.52 ± 16.40	47.69 ± 13.45	<0.001
Men (*n*, %)	15(48.4)	9 (27.3)	0.081
VAS score	5.26 ± 1.90	1.06 ± 2.58	<0.001

### 3.2 The regional brain change in patient group

**Figure 1:**
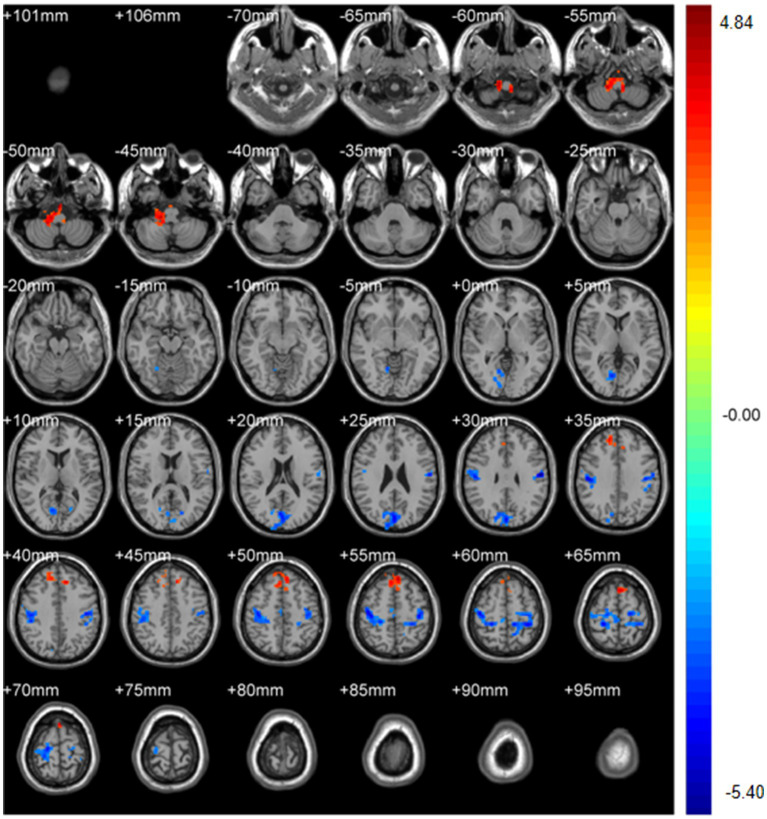
mALFF analysis. Two-sample t-test results are presented. Areas in red indicate significantly increased mALFF value. Areas in blue indicate significantly decreased mALFF value. In the comparison of mALFF value between patient group compared to control group showed significantly increased mALFF in Cerebellum_9_R and right Frontal_Sup_Medial_R.

**Figure 2:**
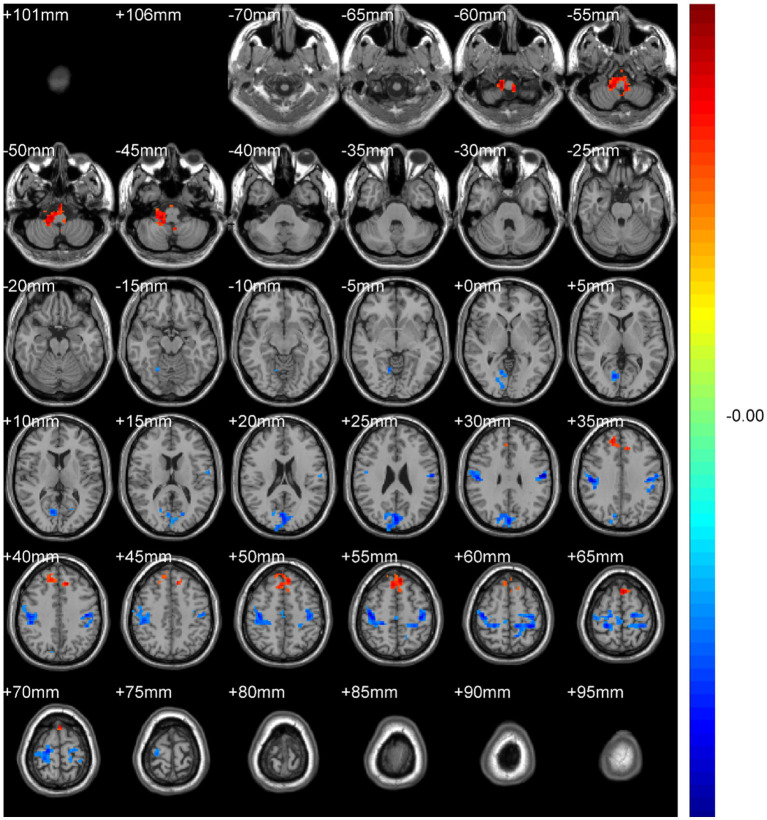
zALFF analysis. Two-sample t-test results are presented. Areas in red indicate significantly increased zALFF value. Areas in blue indicate significantly decreased zALFF value. In the comparison of zALFF value between patient group compared to control group showed significantly increased zALFF in Cerebellum_9_R and right Frontal_Sup_Medial_R.

Regional spontaneous activity (mALFF / zALFF) (Tables 2, 3; Figures 1, 2): Relative to controls, patients showed higher mALFF in the right cerebellar lobule IX (MNI − 6, −39, −57; cluster = 276; *t* = 4.8383) and right medial superior frontal gyrus (MNI − 21, 12, 33; cluster = 465; *t* = 4.2789). Lower mALFF emerged in the bilateral postcentral gyrus [left: MNI − 57, −12, 30; cluster = 501; *t* = −5.3963; right: MNI 48, −21, 36; cluster = 695; *t* = −4.957], right cuneus (MNI 0, −84, 27; cluster = 443; *t* = −4.5173), and right middle temporal gyrus (MNI 57, −57, 6; cluster = 77; *t* = −3.8437). The zALFF map reproduced this pattern: increased activity in right cerebellar lobule IX (MNI − 6, −39, −57; cluster = 310; *t* = 4.8195) and right medial superior frontal gyrus (MNI − 6, 39, 54; cluster = 405; *t* = 4.1027), and decreased activity in the bilateral postcentral gyrus [left: MNI − 57, −12, 30; cluster = 534; *t* = −5.6379; right: MNI 42, −18, 54; cluster = 730; *t* = −5.1794] and right cuneus (MNI 0, −84, 27; cluster = 443; *t* = −4.7479).

**Table 2:** mALFF differences.

Brain regions	Hemisphere	Cluster size	Cluster centroid MNI Coordinates			t-value
			X	Y	Z	
Cerebellum_9	R	276	-6	−39	−57	4.8383
Cuneus	R	443	0	−84	27	−4.5173
Temporal_Mid	R	77	57	−57	6	−3.8437
Postcentral	L	501	−57	−12	30	−5.3963
Postcentral	R	695	48	−21	36	−4.957
Frontal_Sup_Medial	R	465	−21	12	33	4.2789

**Table 3:** zALFF differences.

Brain regions	Hemisphere	Cluster size	Cluster centroid MNI Coordinates			t-value
			X	Y	Z	
Cerebellum_9	R	310	−6	−39	−57	4.8195
Cuneus	R	443	0	−84	27	−4.7479
Postcentral	L	534	−57	−12	30	−5.6379
Postcentral	R	730	42	−18	54	−5.1794
Frontal_Sup_Medial	R	405	−6	39	54	4.1027

**Figure 3:**
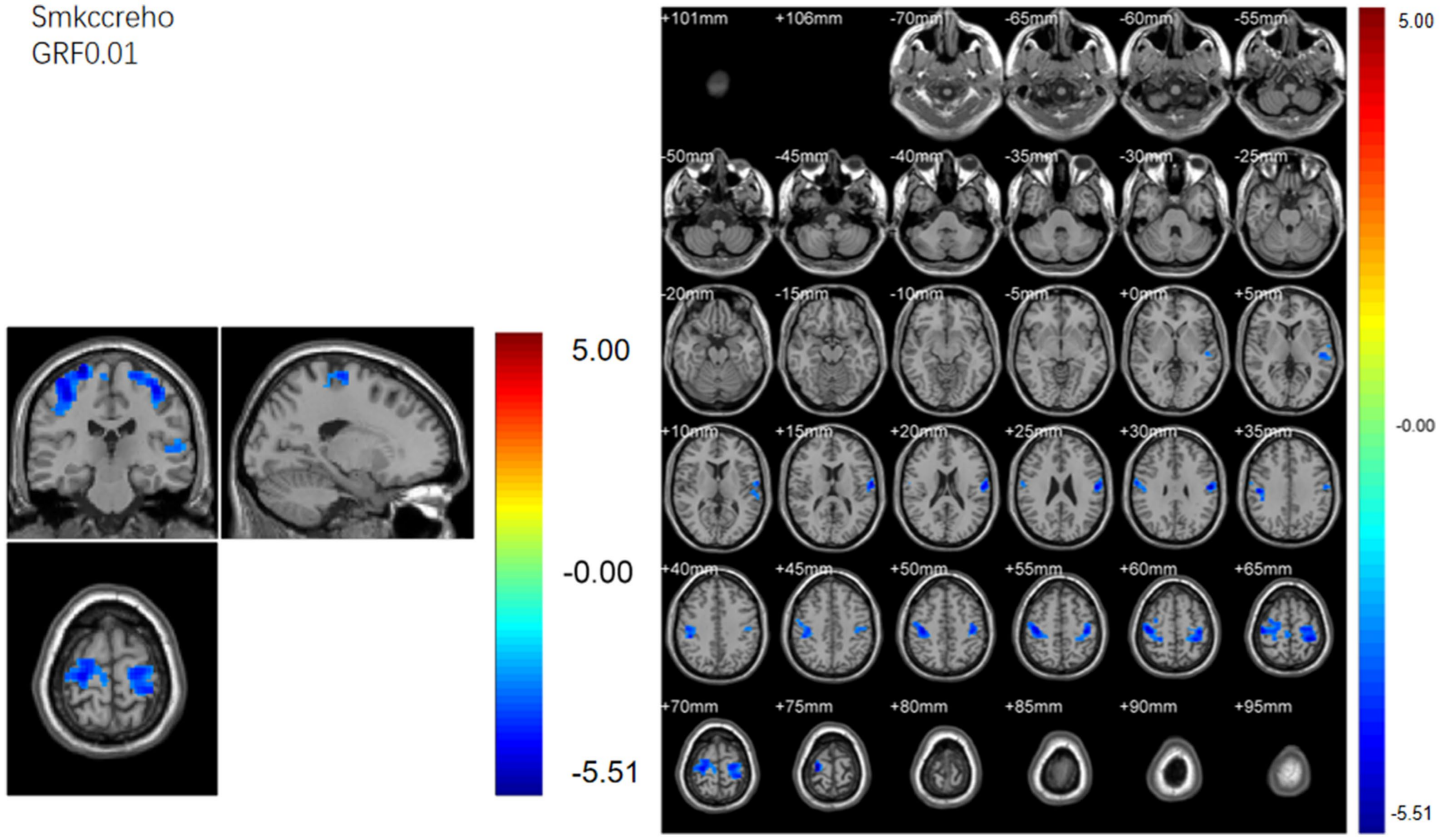
SMKCCREHO analysis. Two-sample t-test results are presented. Areas in blue indicate significantly decreased SMKCCREHO value. In the comparison of SMKCCREHO value between patient group compared to control group showed significantly decreased SMKCCREHO in right and left Postcentral gyus.

Regional homogeneity (ReHo) (Table 4; Figure 3): Using the SMKCC method, ReHo decreased in the left postcentral gyrus (MNI − 57, −12, 27; cluster = 641; *t* = −5.2072) and right postcentral gyrus (MNI 27, −21, 75; cluster = 680; *t* = −5.5139).

**Table 4:** ReHo differences (SMKCC method).

Brain regions	Hemisphere	Cluster size	Cluster centroid MNI Coordinates			t-value
			X	Y	Z	
Postcentral	L	641	−57	−12	27	−5.2072
Postcentral	R	680	27	−21	75	−5.5139

### 3.3 The whole brain changes in patient group

**Figure 4:**
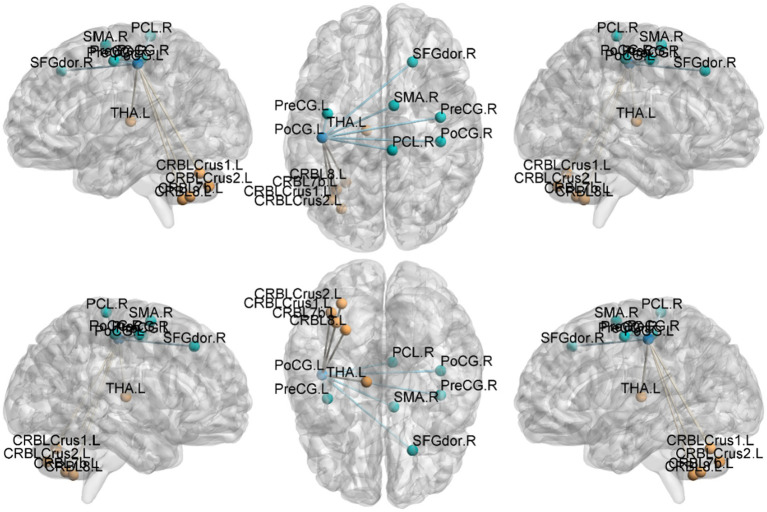
Functional connection with the left Postcentral gyrus as the seed point for patient group compared to control group. The deep blue spheres represent regions of interest, the light blue spheres represent brain regions with decreased functional connectivity to the regions of interest, and the orange spheres represent brain regions with increased functional connectivity to the regions of interest.

**Figure 5:**
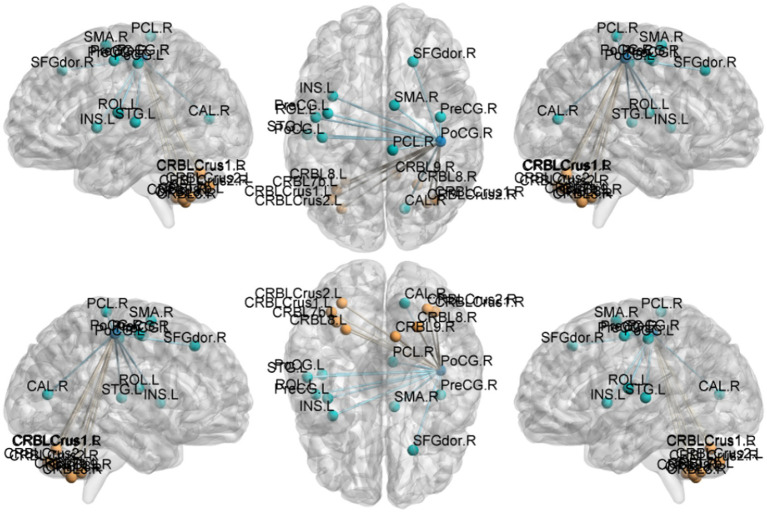
Functional connection with the right Postcentral gyrus as the seed point for patient group compared to control group. The deep blue spheres represent regions of interest, the light blue spheres represent brain regions with decreased functional connectivity to the regions of interest, and the orange spheres represent brain regions with increased functional connectivity to the regions of interest.

**Figure 6:**
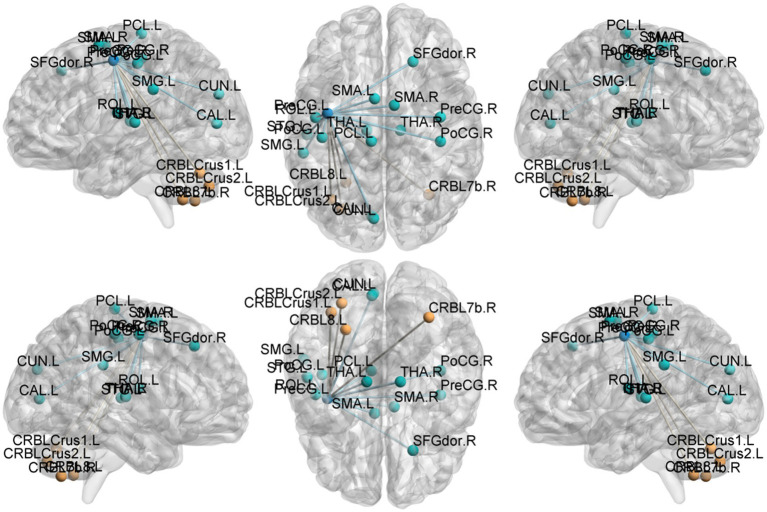
Functional connection with the left Precentral gyrus as the seed point for patient group compared to control group. The deep blue spheres represent regions of interest, the light blue spheres represent brain regions with decreased functional connectivity to the regions of interest, and the orange spheres represent brain regions.

**Figure 7:**
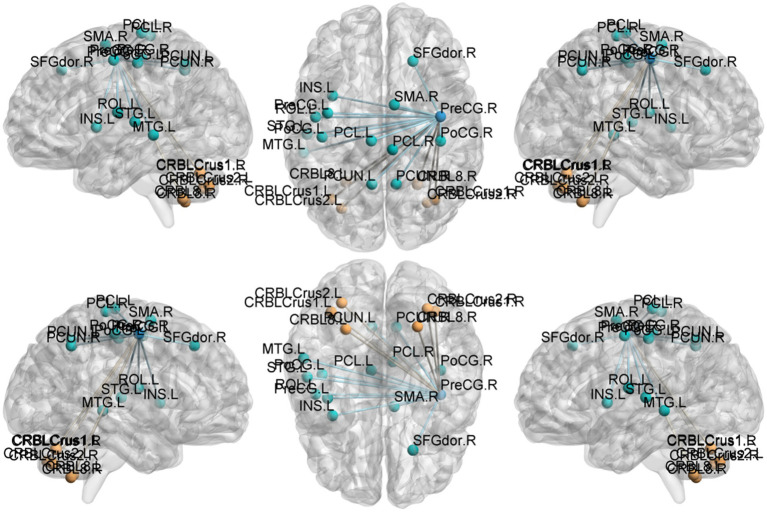
Functional connection with the right Precentral gyrus as the seed point for patient group compared to control group. The deep blue spheres represent regions of interest, the light blue spheres represent brain regions with decreased functional connectivity to the regions of interest, and the orange spheres represent brain regions with increased functional connectivity to the regions of interest.

Seed-based functional connectivity (FC) (Tables 5–8; Figures 4–7):

**Table 5:** Functional connection with the left postcentral gyrus as the seed point for patient group compared to subjects with control group.

Brain regions	Hemisphere	Cluster size	Cluster centroid MNI Coordinates			t-value
			X	Y	Z	
Cerebelum_Crus1	L	131	−42	−63	−36	4.4727
Thalamus	L	67	−18	−21	12	4.1848
Precentral	R	104	39	−15	51	−4.1961
Postcentral	L	113	−27	−33	66	−4.1962
Paracentral_Lobule	R	160	9	−21	69	−4.3763

**Table 6:** Functional connection with the right Postcentral gyrus as the seed point for patient group compared to subjects with control group.

Brain regions	Hemisphere	Cluster size	Cluster centroid MNI Coordinates			*t*-value
			X	Y	Z	
Cerebelum_Crus2	L	129	−30	−57	−45	4.4861
Cerebelum_Crus1	R	127	15	−51	−45	4.6384
Temporal_Sup	L	197	−54	−12	27	−5.1077
Calcarine	R	76	12	−51	0	−3.9785
Precentral	R	316	39	−15	54	−5.2055
Postcentral	L	137	−45	−15	54	−4.4583

**Table 7:** Functional connection with the left precentral gyrus as the seed point for patient group compared to control group.

Brain regions	Hemisphere	Cluster size	Cluster centroid MNI Coordinates			t-value
			X	Y	Z	
Cerebelum_Crus2	L	198	−42	−60	−39	4.495
Temporal_Sup	L	115	−45	−18	12	−4.5627
Thalamus	R	58	15	−15	12	4.5659
Cuneus	L	63	−9	−84	24	−4.0804
Thalamus	L	55	−15	−21	12	4.9435
Postcentral	L	321	−39	−27	57	−4.7458
Postcentral	R	143	36	−36	60	−4.6398
Frontal_Sup	R	108	27	−24	75	−4.233

**Table 8:** Functional connection with the right precentral gyrus as the seed point for patient group compared to control group.

Brain regions	Hemisphere	Cluster size	Cluster centroid MNI Coordinates			t-value
			X	Y	Z	
Cerebelum_Crus1	R	235	30	−75	−36	5.0247
Cerebelum_Crus2	L	214	−3	−69	−30	4.685
Temporal_Sup	R	200	−45	−18	12	−5.1304
Postcentral	L	186	−42	−18	51	−4.0706
Postcentral	R	450	42	−18	54	−5.0403
Precuneus	R	110	−3	−45	57	−4.5909

Postcentral gyrus seeds: Left postcentral seed: showed stronger FC with left cerebellar Crus I (MNI − 42, −63, −36; cluster = 131; *t* = 4.4727) and left thalamus (MNI − 18, −21, 12; cluster = 67; *t* = 4.1848); showed weaker FC with right precentral gyrus (MNI 39, −15, 51; cluster = 104; *t* = −4.1961), left postcentral gyrus (MNI − 27, −33, 66; cluster = 113; *t* = −4.1962), and right paracentral lobule (MNI 9, −21, 69; cluster = 160; *t* = −4.3763). Right postcentral seed: showed stronger FC with left cerebellar Crus II (MNI − 30, −57, −45; cluster = 129; *t* = 4.4861) and right cerebellar Crus I (MNI 15, −51, −45; cluster = 127; *t* = 4.6384); showed weaker FC with left superior temporal gyrus (MNI − 54, −12, 27; cluster = 197; *t* = −5.1077), right calcarine cortex (MNI 12, −51, 0; cluster = 76; *t* = −3.9785), right precentral gyrus (MNI 39, −15, 54; cluster = 316; *t* = −5.2055), and left postcentral gyrus (MNI − 45, −15, 54; cluster = 137; *t* = −4.4583).

Precentral gyrus seeds: Left precentral seed: showed stronger FC with left cerebellar Crus II (MNI − 42, 60, −39; cluster = 198; *t* = 4.495) and with the thalamus bilaterally [right thalamus (MNI 15, −15, 12; cluster = 58; *t* = 4.5659) and left thalamus (MNI − 15, −21, 12; cluster = 55; *t* = 4.9435)]; showed weaker FC with left superior temporal gyrus (MNI − 45, 18, 12; cluster = 115; *t* = −4.5627), left cuneus (MNI − 9, −84, 24; cluster = 63; *t* = −4.0804), left postcentral gyrus (MNI − 39, −27, 57; cluster = 321; *t* = −4.7458), right postcentral gyrus (MNI 36, −36, 60; cluster = 143; *t* = −4.6398), and right superior frontal gyrus (MNI 27, −24, 75; cluster = 108; *t* = −4.233). Right precentral seed: showed stronger FC with right cerebellar Crus I (MNI 30, −75, −36; cluster = 235; *t* = 5.0247) and left cerebellar Crus II (MNI − 3, −69, −30; cluster = 214; *t* = 4.685); showed weaker FC with right superior temporal gyrus (MNI − 45, −18, 12; cluster = 200; *t* = −5.1304), left postcentral gyrus (MNI − 42, −18, 51; cluster = 186; *t* = −4.0706), right postcentral gyrus (MNI 42, −18, 54; cluster = 450; *t* = −5.0403), and right precuneus (MNI − 3, −45, 57; cluster = 110; *t* = −4.5909).

**Figure 8:**
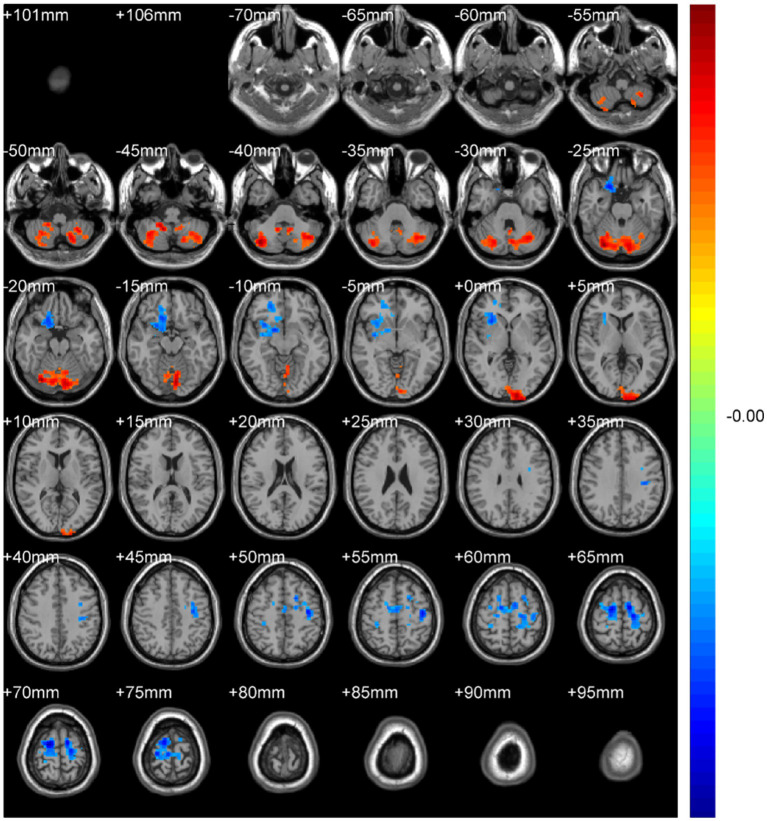
DegreeCentrality(Bi-SmDegreeCentrality). Areas in blue indicate significantly decreased value,areas in red indicate significantly increased value.

**Figure 9:**
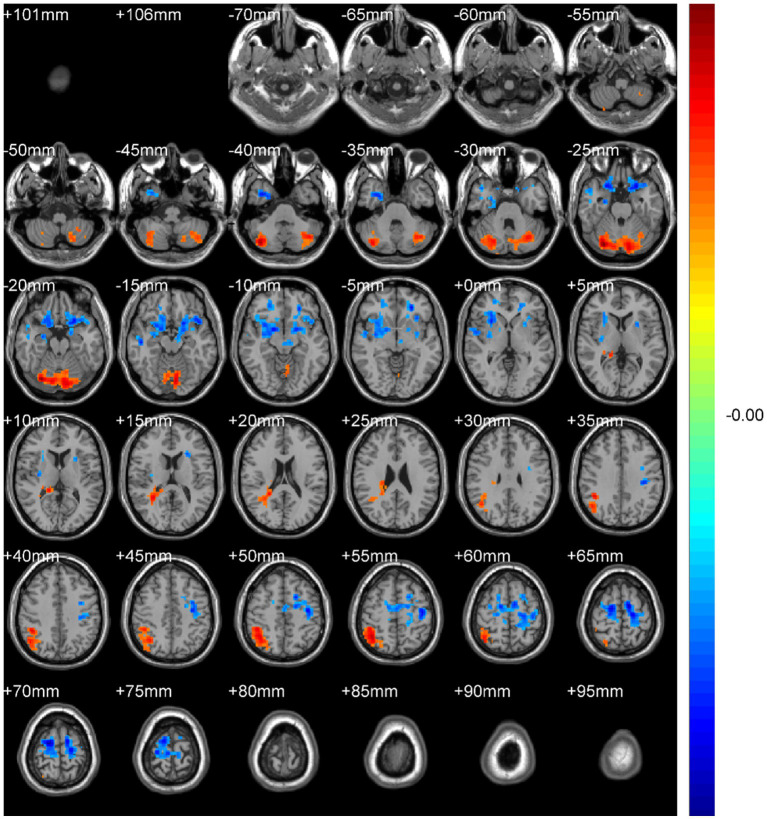
DegreeCentrality(Bi-SzDegreeCentrality). Areas in blue indicate significantly decreased value,areas in red indicate significantly increased value.

**Figure 10:**
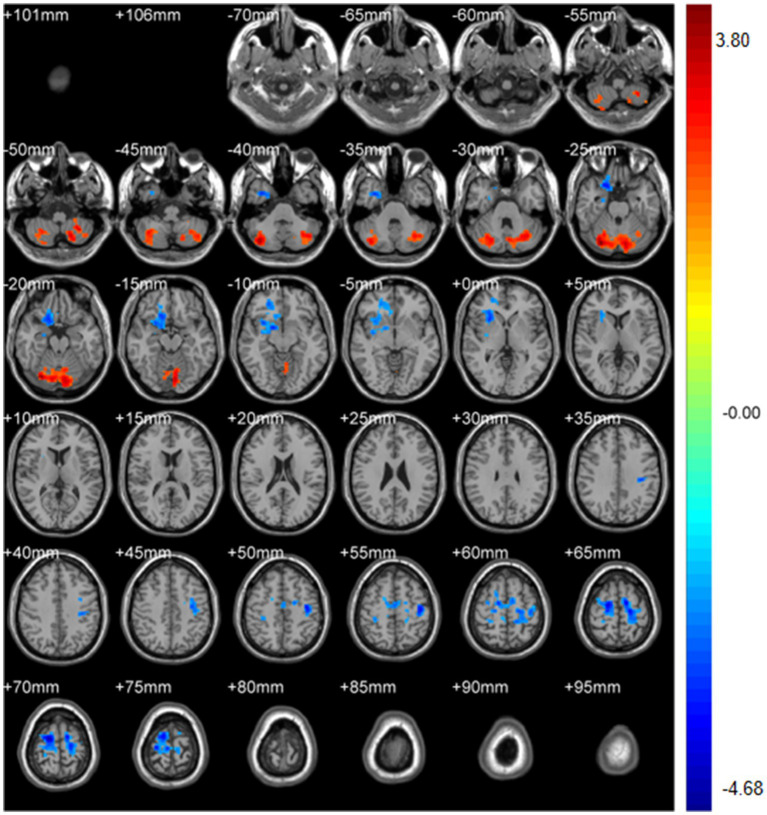
DegreeCentrality(weighted-SmDegreeCentrality). Areas in blue indicate significantly decreased value,areas in red indicate significantly increased value.

**Figure 11:**
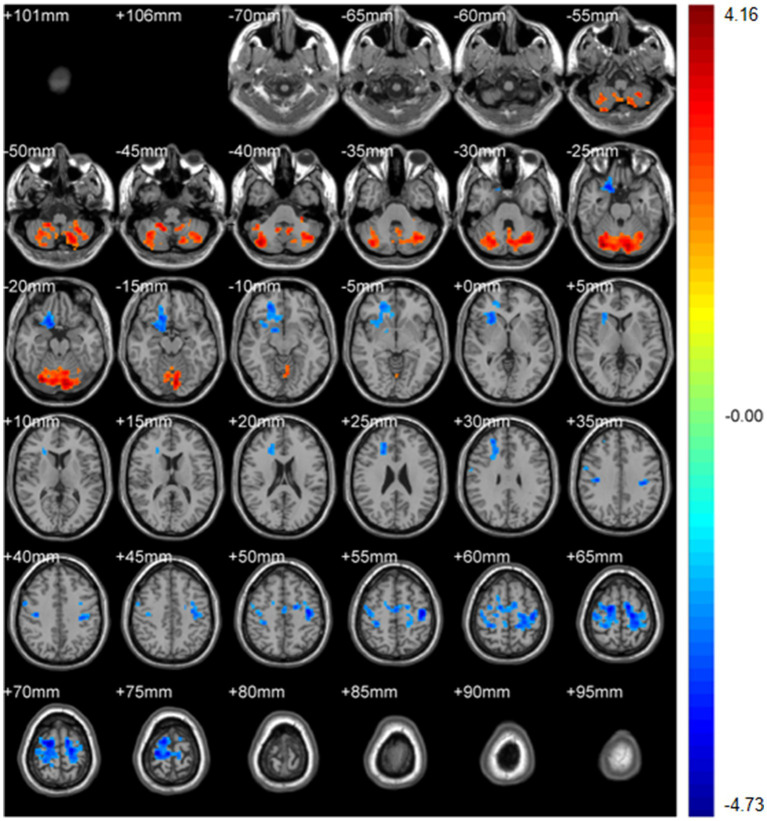
DegreeCentrality(weighted-SzDegreeCentrality). Areas in blue indicate significantly decreased value,areas in red indicate significantly increased value.

Degree centrality (DC) (Tables 9–12; Figures 8–11): Across DC variants, cerebellar Crus I/II showed increased degree centrality, whereas motor and orbitofrontal hubs showed decreased degree centrality.

**Table 9:** DegreeCentrality (Bi-SmDegreeCentrality).

Brain regions	Hemisphere	Cluster size	Cluster centroid MNI Coordinates			t-value
			X	Y	Z	
Cerebelum_Crus1	L	1724	30	−75	−21	3.9172
Frontal_Sup_Orb	R	599	21	21	−27	−3.7685
Precentral	L	1,064	15	−9	69	−4.7716

**Table 10:** DegreeCentrality (Bi-SzDegreeCentrality).

Brain regions	Hemisphere	Cluster size	Cluster centroid MNI Coordinates			t-value
			X	Y	Z	
Cerebelum_Crus1	L	1,171	30	−75	−24	4.2187
Putamen	R	1,021	21	21	−27	−3.9621
Frontal_Inf_Orb	L	594	−21	18	−24	−3.8329
Angular	R	781	42	−54	54	3.6977
Precentral	L	1,277	15	−9	69	−4.8323

**Table 11:** DegreeCentrality (weighted-SmDegreeCentrality).

Brain regions	Hemisphere	Cluster size	Cluster centroid MNI coordinates			*t*-value
			X	Y	Z	
Cerebelum_Crus1	L	1,250	30	−75	−24	3.804
Frontal_Sup_Orb	R	690	21	21	−27	−3.8201
Precentral	L	1,037	18	−9	69	−4.6758

**Table 12:** DegreeCentrality (weighted-SzDegreeCentrality).

Brain regions	Hemisphere	Cluster size	Cluster centroid MNI Coordinates			t-value
			X	Y	Z	
Cerebelum_Crus1	L	1947	−6	−81	−18	4.1556
Frontal_Sup_Orb	R	732	21	21	−27	−3.7573
Precentral	L	1,633	18	−9	69	−4.7346

Binary-SmDegreeCentrality: left Crus I (MNI 30, −75, −21; cluster = 1,724; *t* = 3.9172) showed increased degree centrality; right superior orbital frontal gyrus (MNI 21, 21, −27; cluster = 599; *t* = −3.7685) and left precentral gyrus (MNI 15, −9, 69; cluster = 1,064; *t* = −4.7716) showed decreased degree centrality.

Binary-SzDegreeCentrality: left Crus I (MNI 30, −75, −24; cluster = 1,171; *t* = 4.2187) and right Angular gyrus (MNI 42, −54, 54; cluster = 781; *t* = 3.6977) showed increased degree centrality; right Putamen (MNI 21, 21, −27; cluster = 1,021; *t* = −3.9621), left inferior orbital frontal gyrus (MNI − 21, 18, −24; cluster = 594; *t* = −3.8329) and left precentral gyrus (MNI 15, −9, 69; cluster = 1,277; *t* = −4.8323) showed decreased degree centrality.

Weighted-SmDegreeCentrality: left Crus I (MNI 30, −75, −24; cluster = 1,250; *t* = 3.8040) showed increased degree centrality; right superior orbital frontal gyrus (MNI 21, 21, −27; cluster = 690; *t* = −3.8201) and left precentral gyrus (MNI 18, −9, 69; cluster = 1,037; *t* = −4.6758) showed decreased degree centrality.

WeightedSzDegreeCentrality: left Crus I (MNI − 6, −81, −18; cluster = 1,947; *t* = 4.1556) showed increased degree centrality; right superior orbital frontal gyrus (MNI 21, 21, −27; cluster = 732; *t* = −3.7573) and left precentral gyrus (MNI 18, −9, 69; cluster = 1,633; *t* = −4.7346) showed decreased degree centrality.

## 4 Discussion

Across complementary resting-state metrics—regional activity (mALFF/zALFF), local synchrony (ReHo), pairwise coupling (seed-based FC), and graph metrics (degree centrality, DC)—patients with spinal diseases–related pain exhibit a coherent reorganization of the sensorimotor–thalamo–cerebellar system. Convergent evidence indicates (i) down-regulation within S1/M1, reflected by lower ALFF/zALFF, reduced ReHo, diminished DC, and weaker intra-sensorimotor FC; and (ii) up-weighting of cerebellar nodes, including increased ALFF/zALFF in Cerebellum lobule IX and consistently elevated DC in Crus I/II across binary and weighted thresholds. Beyond the primary motor system, reduced coupling with superior temporal gyrus, calcarine/cuneus, and precuneus/DMN suggests broader consequences for auditory–temporal integration, early visual processing, and default-mode subsystems. Laterality was modest overall in the network-level synthesis, though the voxelwise analyses highlight pronounced right lobule IX hyperactivity (ALFF t = 4.84) and left Crus I hyperconnectivity (DC peak *t* = 4.22), nominating postcentral and cerebellar clusters as hubs in a shift from cortical sensorimotor dominance toward cerebellar–subcortical coordination.

The combined pattern is compatible with sensorimotor dysrhythmia and compensatory gating models in chronic pain. The dual-mode cerebellar signature suggests subregional dissociation: lobule IX may contribute to more direct nociceptive integration (34), whereas Crus I appears to participate in compensatory network reorganization via enhanced thalamo-cortical coupling (35). These observations align with literature on cerebellar involvement in pain anticipation (36) and descending modulatory control (37). The prefrontal findings indicate functional segregation within medial PFC, with anterior (*t* = 4.28) versus posterior (*t* = 4.10) subregions showing differential activation that plausibly map onto the affective (38) and cognitive-evaluative (39) dimensions of pain, respectively, and thus motivate subregion-specific modulation strategies. Meanwhile, the preserved thalamic coupling (e.g., left thalamus *t* = 4.94) in the context of cortical hypoactivity is consistent with roles proposed for central sensitization (40) and enhanced nociceptive relay (41). Notably, age effects were negligible in these data, in keeping with reports of minimal association between age and clinical pain perception in similar cohorts (42, 43), suggesting the observed signatures are primarily symptom-related rather than age-driven.

Together, these results nominate cerebellar Crus I/II and sensorimotor–thalamic loops as testable targets for neuromodulation or rehabilitation. In particular, Crus I/II DC and cerebello–S1/M1 FC emerge as plausible network-level readouts for patient stratification and treatment monitoring. The observed prefrontal subspecialization further implies subregion-specific stimulation or neurofeedback protocols tailored to affective versus cognitive pain components.

Limitations: This work is retrospective with a modest sample size, limiting causal inference and external generalizability. Clinical heterogeneity (spinal pathology, medication, pain duration/treatment history) may introduce variance beyond modeled covariates. Results are group-level and not individually predictive. Graph metrics such as DC can be sensitive to thresholding and pipeline parameters; although convergence across binary and weighted thresholds increases confidence, absolute DC values warrant cautious interpretation. Despite stringent motion controls, residual micromovements and state factors (attention, medication) cannot be fully excluded. Finally, the absence of behavioral correlations (sensorimotor performance, detailed pain phenotyping) constrains mechanistic claims.

Future directions: Prospective, phenotype-stratified and longitudinal cohorts with harmonized acquisition and open, standardized pipelines should test the stability, specificity, and prognostic value of these signatures. Interventional designs (neuromodulation, neurofeedback, targeted rehabilitation) can probe causality by tracking Crus I/II DC and cerebello–sensorimotor FC as mechanistic endpoints alongside clinical outcomes. Multimodal integration (structural, diffusion, and task paradigms) and behavioral anchoring will be essential to refine theranostic utility.

## 5 Conclusion

Patients with spinal diseases–related pain show a reproducible, multimodal reconfiguration of resting-state networks: down-regulation of primary sensorimotor cortices and up-weighting of cerebellar nodes (lobule IX, Crus I/II), with strengthened cerebello–sensorimotor and thalamo-cortical coupling and reduced interactions with temporal, occipital, and precuneus/DMN regions. Prefrontal subspecialization further suggests altered evaluative–affective control. While associative, this coherent signature refines the central phenotype as a shift of network load toward cerebellar–subcortical loops and nominates cerebellar (Crus I/II, lobule IX), thalamic, and S1/M1 circuits as testable targets. Network-level readouts—particularly Crus I/II degree centrality and cerebello–S1/M1 connectivity—warrant prospective evaluation as biomarkers for stratification and treatment monitoring.

## Data Availability

The raw data supporting the conclusions of this article will be made available by the authors, without undue reservation.
